# Experimental and simulation results of the adsorption of Mo and V onto ferrihydrite

**DOI:** 10.1038/s41598-018-37875-y

**Published:** 2019-02-04

**Authors:** Loredana Brinza, Hong Phuc Vu, Mariana Neamtu, Liane G. Benning

**Affiliations:** 1 Alexandru Ioan Cuza University of Iasi, Institute for Interdisciplinary Research, Science Research Department, Iasi, 700107 Romania; 2 University of Leeds, School of Earth & Environment, Earth Surface Sciences Inst, Leeds, LS2 9JT West Yorkshire United Kingdom; 3 University of Melbourne, The Faculty of Science, School of Earth Sciences, Melbourne, Victoria 3010 Australia; 4 GFZ German Research Centre for Geosciences, D-14473 Potsdam, Germany; 5 Department of Earth Sciences, Free University of Berlin, 12249 Berlin, Germany

## Abstract

This study aims to highlight discrepancies between experimental and simulation linked to the mechanisms of Mo and V adsorption onto ferrihydrite (FHY) nanoparticles. We have measured adsorption capacities and uptake efficiencies and then fitted and compared these with outputs from various geochemical and adsorption models that were run as a function of pH, surface area (SA) and ferrihydrite particles size distributions. Our results revealed that the experimental data for the Mo system could be fitted very well, but this was not the case for the V system, when a model default value for the SA of FHY of 600 m^2^ g^−1^ was used. The discrepancy in the results for the V system can be explained by the lack of specific V species and/or associated constants in databases and variation in software versions, which change the outputted chemical species. Our comparative results also confirm that any experimental variables used as modelling inputs need to be checked carefully prior to any modelling exercises.

## Introduction

Beside their important role as micronutrients for living organisms^1–4^, Mo and V are considered good paleo environmental proxies^5–9^, but they act also as pollutants as well as catalysts^10,11^. Their interactions with iron oxyhydroxides in surface and polluted waters, seawater and sediments affect their mobility and cycles in these environments. Adsorption, one of the most important interactions taking place at the mineral-water interface, strongly controls the fate and availability of Mo and V.

Ferrihydrite (FHY) is an iron oxyhydroxide often called “amorphous ferric oxide” or “hydrous ferric oxide,” which can be found in different environmental media such as soils, iron-rich or polluted waters (*e.g*., mine tailings and acid mine drainage), deep oceans, lake sediments, hydrothermal vents or hot- and cold-spring deposits^5,12–15^. FHY is often associated with a variety of other amorphous or crystalline minerals^16–21^ and has a well-documented ability to adsorb or co-precipitate many ions including toxic or trace metals^18,20,22–29^. There are two types of FHY: 2-line and 6-line ferrihydrite, differing in water contents, surface areas, particle sizes and naturally crystallinity^18,19^. For 2-line FHY, Hiemstra and Riemsdijk^30^ suggested that single-coordinated surface groups dominate and that these groups are present in two structural configurations: at the edge of a single Fe-octahedron or at a single corner of two adjacent Fe-octahedrons^30^. One of the main characteristics of FHY is its very large surface area (SA). However, yet a common value is not available and a huge range of SA’s (from 120 to 840 m^2^ g^−1^) has been reported for synthetic and natural 2 and 6 line-FHY^19,22,31^. As a result of this high surface area, FHY can adsorb cations (*e.g*., Cd^2+^, Cu^2+^, Pb^2+^, Co^2+^, Mn^2+^, Cr^3+^, Cr^6+^, Ni^2+^, Zn^2+^, Ag^+^), anions (*e.g*., PO_4_^3−^, MoO_4_^2−^, HVO_4_^2−^, VO_2_(OH)_2_^−^, VO_3_(OH)^2−^, WO_4_^2−^) and organics (herbicides: 7 chloro-3-methylquinoline-8-carboxylic acid, thiazafluron; mugineic acid and p-hydroxybenxoic acid)^19,20,32–43^.

Despite the large variations in SA reported, in most sorption modelling codes, the highest values for FYH SA (i.e., 600, 700 or 750 m^2^ g^−1^) are most often used as defaults^15,33,41,44–46^. Furthermore, adsorption of metal species onto oxyhydroxides is often modelled using a variety of surface complexation models: the generalized two layer model called Diffuse Layer Model (DLM), the Constant Capacitance Model (CCM), the CD-Music Layer Model (CDM)^15,33,47,48^ or the Triple Layer Model (TLM)^45,49^. The selection of a particular model for a specific system depends on the chemistry of the system (e.g., metal concentration, ionic strengths, presence of other ions, etc.), the number of assumptions and the experience/expertise of researchers. For example, Dzombak and Morel^30^ recommended the DLM model as the most suitable for modelling ion adsorption onto iron oxyhydroxides^32^, and Gustafsson^33^ created a version of this DML model for hydrous ferric oxides (HFO) using the Visual Minteq code^33,44,50^.

When we started with this current study we realized that simulations of Mo and V adsorption onto FHY as a function of pH were scarce^15,46^, and that those existing had only been done for systems at high ionic strength (IS). For example, Gustafsson^33^ used the DLM and CDM models to fit Mo sorption onto FHY at ionic strength of 0.01 M. The initial fit were not very good and this led to a model optimization by generating new species and appropriate constants (i.e., FeOMo(OH)_5_ with logK = 17.96 for DLM and FeOMo(OH)_5_^0.5^ with logK = 18.28 and FeOMoO_3_^−1.5^ with logK = 11.17 for CDM). Such an optimization, improved the DLM fit and after adding more species (i.e., Fe_2_O_2_MoO^2−^ with logK = 18.71; FeOH_2_^½+^…MoO_4_^2−^ with logK = 11.06) this model could thus be used to model Mo adsorption in onto HFO’s in soils^15,33^. Similarly, using the CDM model for V sorption onto FHY, Larsson *et al*.^46^ aimed to optimize surface complexation constants of V species at the FHY surface. He modelled this for complex systems and at high IS^46^, yet the results revealed that a full understanding of the adsorption is still lacking.

Information about sorption mechanism is often obtained through X-Ray Absorption Spectroscopy (XAS) measurements^15,45,46,51,52^. These studies showed that Mo sorbed to FHY forms an edge sharing bidentate complex at pH of 4.2 and 7.3, and an additional corner-sharing bidentate complex at pH 4.2^15^. XAS analyses of Mo adsorbed onto goethite indicated that with decreasing pH, the Mo (VI) coordination environment changes from a tetrahedral (inner-sphere surface species via corner- and edge-sharing attachment with iron octahedra) to an octahedral coordination. Moreover, under acidic conditions an additional MoO_6_ polymer attached to the octahedral iron suggested the formation of surface Mo-polymers^52^. For the V system, Peacock and Sherman^45^ showed that V adsorbed onto goethite at pH between 3 and 9 as a bidentate, corner-sharing inner sphere complex, and that possibly a VO_2_(O, OH)_2_ edge sharing complex was present at a pH of 8.3^45^. Larsson *et al*.^46^ also found that V adsorbs to FHY at pH values of 3.6, 3.9, 6.5 and 9.39 as an edge-sharing vanadate complex^46^. Despite this seeming plethora of data, the molecular configuration of Mo and V surface complexes adsorbed onto FHY surfaces remain poorly know, and additional studies covering a wider range of environmental conditions is needed in order to better develop surface complexation models.

We try to fill this gap in part by combining detailed experimental measurements of Mo and V adsorption (single ion in solution) onto FHY over the pH range between 4 to 9, over 22 h, and also and low IS (0.001) with modelling using different codes and databases. The results will improve our understanding of Mo and V uptake processes onto FHY.

## Results and Discussions

### Ferrihydrite characteristics

The measured BET surface area of the FHY synthetized in this study was 200 ± 16 m^2^ g^−1^. This value falls within the wide range of SA reported for FHY (between 120 and 840 m^2^ g^−1^). This low value will likely lead to differences in experimental data *vs*. simulations (where 600 m^2^ g^−1^ is the default^33^). Our potentiometric titrations (Table S1; Supplementary Information, SI) revealed a FHY point of zero charge (PZC) of 7.96, a result in agreement with previous literature findings^24,25^ which indicates that below this pH the FHY surface is positively charged whereas above this pH the surface is negatively charged. Particle/aggregate size measurements by dynamic light scattering (DLS) showed that FHY was present as large aggregates in solution with aggregate sizes ranging between 3 and 150 µm. The average was 75 µm and this size and a single 2-line FHY particle size previously determined by TEM of 4 nm^53^ will be used in the simulations. Full details of all FHY characterizations are presented in the SI.

### Mo and V adsorption: pH effects and kinetics

**Figure 1:**
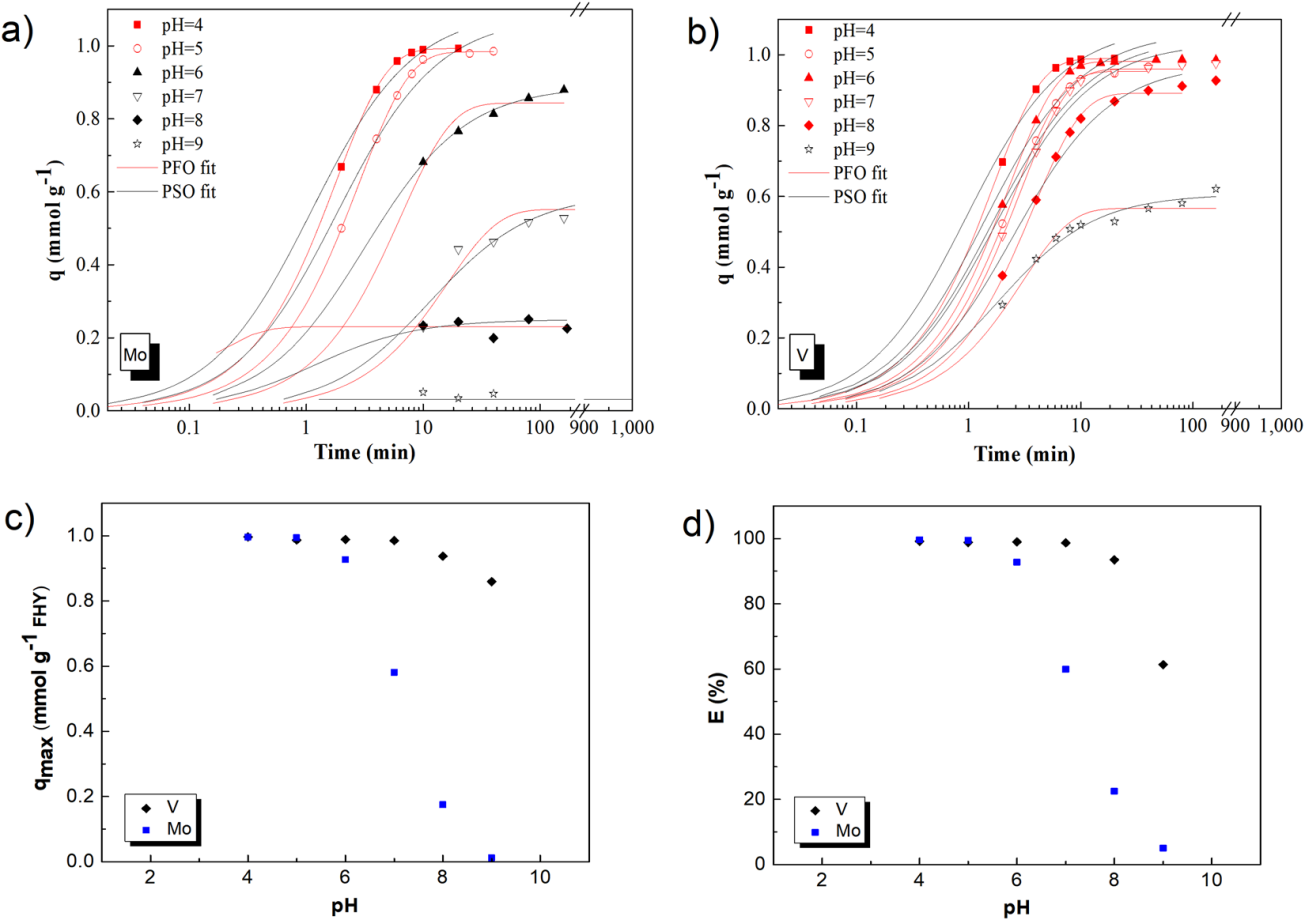
The kinetics of molybdenum (**a**) and vanadium (**b**) adsorption onto ferrihydrite at different pH values in the mono-sorbate systems. Symbols represent experimental data, and lines show the fits to a pseudo-first (PFS) and - second (PSO) order kinetic model. The influence of pH on molybdenum and vanadium adsorption onto ferrihydrite is also shown: the experimental results are expressed as uptake capacity (**c**) and removal efficiency (**d**). Experimental conditions: C_i Mo/V_ = 100 µM metal and C_FHY_ = 0.1 gL^−1^). Errors in metal concentrations were smaller than the size of the symbols.

Mo and V loadings on FHY, expressed as uptake capacities (q) and removal efficiencies (E, kinetics discussed in the SI, Fig. S2) are plotted in Fig. 1. The results reveal that for both metals the uptake capacities and removal efficiencies follow similar trends, reaching equilibrium values after ~20 minutes and decreasing in both cases with increasing pH.

For both Mo and V, the derived kinetic parameters are strongly dependent on pH and a faster and greater uptake occurred at lower pH, while a slower and lower sorption occurred at a pH near and above the PZC. These trends matched the uptake-pH profiles (Fig. 1c and d). The experimental data showed that sorption occurred after a short induction time (10–20 min). The data were fitted with pseudo-first (PFO) and pseudo-second (PSO) order kinetic models (Fig. 1)^54–56^ and in Table 1 we summarized the kinetic parameters obtained from the fitting. For the Mo system, the experimental data at pH 4 and 5 were fitted very well by the PFO kinetic model, while the data at pH 6 and 7 were better described by the PSO kinetic model. The R-squared for fitting data at pH above 8 was low, suggesting that the employed models are unsuitable. For the V system, the PFO kinetic model fitted very well the data over the pH interval between 4 and 8, whereas the PSO kinetic model better described the adsorption at pH 9 (R^2^ = 0.990 for PSO as opposed to R^2^ = 0.977 for PFO, Table 1). Similar outputs were obtained, when all experimental data were fitted (see Table S2, for comparison).

**Table 1:** Summary of the pseudo-first order (PFO) and pseudo-second order (PSO) kinetic model parameters of Mo and V adsorption onto FHY; best fits highlighted by statistical parameters^*^ (here R^2^ and Adj R^2^ values) marked in bold.

PFO						PSO					N
Mo/pH	q (mmol g^−1^)	k1 (min^−1^)	R^2^	Adj. R^2^	AAD	q (mmol g^−1^)	k2 (g mmol^−1^ min^−1^)	R^2^	Adj. R^2^	AAD	
4	0.992 ± 0.001	0.553 ± 0.004	**0.9999**	**0.9999**	0.123	1.095 ± 0.003	0.824 ± 0.160	0.993	0.991	0.118	7
5	0.983 ± 0.002	0.354 ± 0.003	**0.9998**	**0.9998**	0.103	1.080 ± 0.037	0.507 ± 0.102	0.984	0.981	0.107	8
6	0.843 ± 0.017	0.154 ± 0.019	0.9927	0.9908	0.057	0.889 ± 0.005	0.358 ± 0.021	**0.999**	**0.9995**	0.011	6
7	0.551 ± 0.019	0.061 ± 0.009	0.968	0.9628	0.025	0.594 ± 0.022	0.148 ± 0.033	**0.974**	**0.9705**	0.036	8
8	0.214 ± 0 011	48.440	0.867	0.848	0.0004	0.214 ± 0.013	1.3*10^−28^	0.867	0.848	0.0004	9*
9	0.031 ± 0.006	53.0370	0.376	0.251	0.001	0.031 ± 0.007	1.9*10^−46^	0.376	0.251	0.001	7*
**V/pH**	**q (mmol g** ^**−1**^ **)**	**k1(min** ^**−1**^ **)**	**R** ^**2**^	**Adj. R** ^**2**^	**AAD**	**q (mmol g** ^**-1**^ **)**	**k2(g mmol** ^**−1**^ **min** ^**−1**^ **)**	**R** ^**2**^	**Adj. R** ^**2**^	**AAD**	
4	0.989 ± 0.000002	0.601 ± 0.000009	**1**	**1**	0.116	1.07 ± 0.029	0.978 ± 0.197	0.993	0.992	0.109	7
5	0.953 ± 0.003	0.394 ± 0.005	**0.9997**	**0.9996**	0.092	1.048 ± 0.031	0.588 ± 0.108	0.988	0.986	0.099	8
6	0.981 ± 0.001	0.442 ± 0.0029	**0.9999**	**0.9908**	0.076	1.062 ± 0.027	0.672 ± 0.12	0.989	0.987	0.086	8
7	0.959 ± 0.003	0.351 ± 0.004	**0.9996**	**0.9996**	0.058	1.034 ± 0.028	0.563 ± 0.098	0.984	0.982	0.070	9
8	0.891 ± 0.006	0.267 ± 0.007	**0.9986**	**0.9984**	0.068	0.973 ± 0.025	0.411 ± 0.057	0.988	0.986	0.081	9
9	0.566 ± 0.012	0.332 ± 0.033	0.980	0.977	0.019	0.606 ± 0.010	0.898 ± 0.101	**0.991**	**0.990**	0.028	10

*R^2^, Adj. R^2^, average absolute deviation (AAD) and the residuals of the fit are statistic parameters based on which the choice of which model better fits the experimental data was done. R^2^ and Adj. R^2^ parameters were needed to differentiate between the best fits of scenarios in which different and all numbers of data points were fitted, respectively. R^2^ allows comparison between PFO and PSO kinetic models with different numbers of data points – i.e., time when equilibrium was reached but not including the equilibrium platter (varied function of pH). Adj.R^2^ allows comparison between the models when the same number of data points were considered – i.e., data including equilibrium (see also Table S2 in SI). Where the R^2^ and the Adj. R^2^ parameters were very similar, then the minimal value of AAD and the uniformity of residual profile were used to decide the best fit of the model. Plots of the data fitted up to equilibrium and the obtained associated parameters are presented above, whereas the results from fitting all the data points at equilibrium are displayed for comparison in the SI.

For the two systems, two main components are considered as: Mo/V ions in solution and functional groups at the FHY surface. Whereas the first component is set as a constant (matching geochemical modeling), the second component changes with/if there is a change in pH (as seen in our potentiometric titration results, Table S1). At low pH, the protonated sites on the FHY surface are in excess, allowing the anions to bind quickly, thus following a PFO kinetic path via chemisorption. As pH increases towards the PZC, the number of positive charged reactive sites decreased, thus adsorption efficiency also decreases and a PSO kinetic path prevails, which implies physisorption. The results also reveal that V adsorbed onto the FHY surface over a larger range in pH compared to Mo, an observation that can be explained by the difference in the anions’ valence and deprotonation stages. Vanadium, with three deprotonation stages, could have more available vanadate species to bind onto the FHY surface over the tested pH range. However, as we show later, such a stoichiometric mass balance based argument is not supported by the geochemical modelling results.

In terms of Mo and V removal (Fig. 1d) the data reveal that at pH below 7, V was almost fully removed from solution, yet this efficiency decreased at pH 9 (down to 60%). Mo was almost fully removed from solution at a pH below 6 and its removal efficiency decreased to 60% at pH 7, 22% at pH 8, and 5% at pH 9. For V, at a pH above 7 the removal efficiency profile has a sharper decrease compared to its uptake capacity profile (Fig. 1c) a difference likely a consequence of the fact that the uptake capacity takes into account the adsorbent characteristics, whereas the removal efficiency is a pure expression of metal removal calculated from metal concentration in the supernatant.

Comparing our data to previous studies on Mo^33^ and V^46^ sorption revealed similar trends. For example, for V, our data matches well with the data in Naeem *et al*.^57^, especially as the properties of the FHY they used were similar to ours (SA = 231 m^2^ g^−1^, PZC = 8), and the experiments were conducted under similar experimental conditions (i.e., IS 0.01; ca. 1 mM V and 1.4 g L^−1^ dry mass)^57^. Another V adsorption study carried out by Trefry and Metz^6^ showed that 80% of the V was removed by 2 gL^−1^ FHY within two minutes from synthetic seawater (pH 8 and IS 0.7) that was spiked with 200 µM V. Our experimental data, obtained at much lower IS (averaging 0.001, represent important additions that combined with the other new data will be used in the optimisation of sorption models.

**Figure 2:**
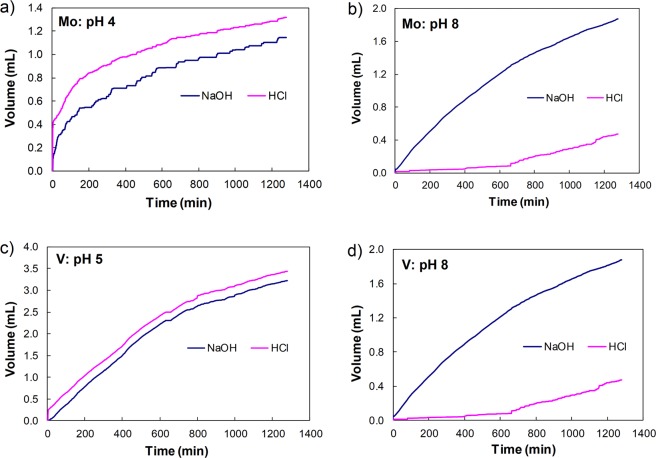
The profiles of hydrochloric acid (0.1 N) (pink line) and sodium hydroxide (0.1 N) (blue line) added to the molybdenum (**a** and **b**) and vanadium (**c** and **d**) absorption systems during their adsorption onto ferrihydrite at low pH (4 for molybdenum, (**a**) and 5 for vanadium, (**c**)) and high pH (8 for both anions, (**b** and **d**)).

When the adsorption behaviour was tested at constant pH (pH 4, 5 and 8; Fig. 2) the data revealed that at low pH, in both systems, slightly more acid was needed as the anions were adsorbed onto the protonated FHY surfaces (Fig. 2a and c). In contrary at high pH more base (1.7 mL for Mo and 3.2 ml for V, at pH 8) than acid was required for the system to maintain the constant pH during the adsorption Fig. 2b and d). The acid and base addition led to a final IS, averaging a value of 0.0012 for V and 0.0005 for Mo systems.

The difference in acid and base volumes added during the adsorption of the two anions are likely related to anion size and valence, which control the adsorption bonding environment and support the potentiometric titration and pH dependent adsorption behaviour for Mo and V (Fig. 1).

Qualitatively, this indirect approach of evaluating sorption mechanisms confirms that at a pH below the PZC, anion adsorption onto the protonated FHY surface occurred with hydroxyl release, whereas, at high pH lower adsorption of bi (for Mo) and tri (for V) valent anions onto the negatively charged FHY surface was associated with release of protons.

### Geochemical modelling of Mo and V speciation *in solution* and their adsorption simulation onto ferrihydrite

Geochemical modelling helped determine speciation of Mo and V in solution that can interact with the FHY surface. Full details about the used codes and databases and assumptions are detailed in the methods and the SI. The results reveal clear differences between the Mo and V speciation modelling outputs from the GW code^58^ (Fig. S1a,c and b,d) and VM^44,50^ codes (Fig. S4e,f). These discrepancies may be due to differences in codes database inputs (e.g., intrinsic constants, species type). We suggest that databases should be checked, compiled and universally formatted so that they can be read and used by all geochemical modelling codes.

**Figure 3:**
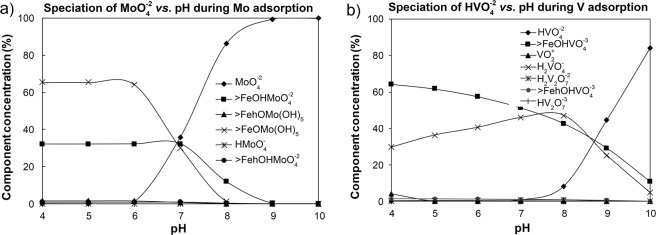
Molybdenum and vanadium surface sites distribution among the dissolved and adsorbed species as a function of pH.

The use of VM code to simulate adsorption as opposed to GW, was done because it contains dedicated models for sorption processes. When the experimental data were compared with the sorption simulation using the VM code^44,50^ Fig. 3), the data showed that free molybdate in solution dominated at a pH above 6, increasing rapidly (to 100%) at pH > 9. At pH < 6, Mo was associated with the FHY surface as FeOMo(OH)_5_ (65%) and FeOHMoO_4_^−2^ (33%) species. The proportions of these complexes decrease with increasing pH, and at pH 7 there were equimolar concentrations of all species. At high pH, free molybdate (MoO_4_^−2^) remained dissolved and not adsorbed. This trend explains the decrease in Mo removal efficiency (to 60%) with increasing pH obtained experimentally (Fig. 1d). Interesting however is the fact that the surface associated Mo species (Table S3) differed markedly between the various databases tested and the molybdate dissociation stoichiometries were also inconsistent among the studied databases.

For the V system (Fig. 3b), the modelling revealed that the percentage of vanadate (HVO_4_^−2^) in solution was low at pH < 8 (<8%) but increased to 45% and 85% at pH 9 and 10, respectively. Thus below pH 8 most of the V in the system is adsorbed onto FHY, supporting the experimental results (see Fig. 1c and d). At pH 4, ~64% of the V species were adsorbed onto FHY as FeOHVO_4_^−3^, whereas 30% was present as the monovalent free vanadate (H_2_VO_4_^−^) species. The importance of the V surface complex, FeOHVO_4_^−3^, decreased with increasing pH, while monovalent vanadate reached a maximum of 50% at pH 8, followed by a sharp decrease at high pH. Thus, at pH 8 only 40% of the total FeOHVO_4_^−3^ species can explain the adsorbed V, while the remaining non-sorbed species are made up of 10% divalent vanadate (HVO_4_^−2^) and 50% monovalent vanadate anion (H_2_VO_4_^−^) (Fig. 3b). These modelling results match well our experimental trends (Fig. 1), yet, quantitatively, the simulation results explain only ~40–50% of experimental data. To explore the causes of this difference we investigated variation in species in the available databases. We tested: (i) values of intrinsic and dissociation constants; (ii) their origin (i.e., reference); (iii) whether the experimental conditions at which the constants were obtained from were similar to the ones in this study, and (iv)what the variations in species parameters are in each database. It became clear that for example the Feo_dlm.mdb database (used in previous version of the VM code) contains additional V surface associated species besides “=FehOHVO_4_^3−^”. These are “=FehH_2_VO_4_”; “=FehHVO_4_^−^”; “=FehVO_4_^2−^”, which correspond to the expected protonation/deprotonation stoichiometry of the reaction in solution and on surfaces. However, these species were excluded in the latest database. Furthermore, different values for the sole surface associated species, “=FehOHVO_4_^3−^”, were found in various databases (i.e., −0.73 in the current database, 16.63 in M4_feo_dlm.vdb database, 13.57 in Dzombak and Morel^30^). Finally, looking for the references cited as the source of these constants (i.e., Cruy 2000 and CruyDrag2002) did reveal that these entries must be incomplete/erroneous as no data or equivalent references could be found.

Combining the species distribution derived from the geochemical modelling and adsorption simulation, with the experimental potentiometric titration results, lead us to conclude that adsorption proceeded as follows: below the PZC of FYH (<8) multi protonated anions bind to the positively charged FHY surfaces likely via inner sphere complexes, while at pH values above the PZC the anions only adsorb weakly, probably via outer sphere complexes.

### Effect of code version and database

**Figure 4:**
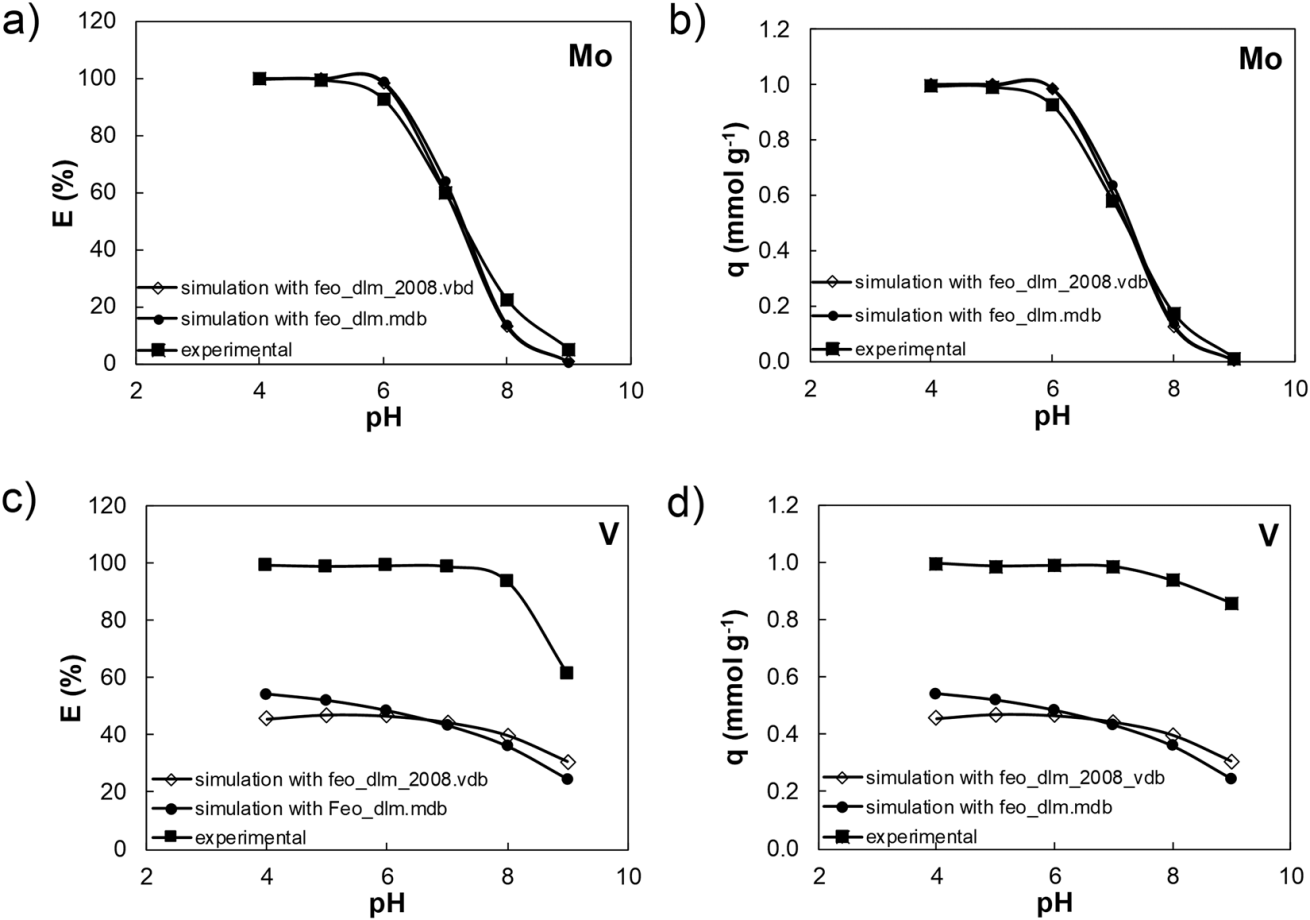
Comparison between the Mo (**a** and **b**) and V (**c** and **d**) experimental data and simulation results (using a surface area 600 m^2^ g^−1^) expressed as E (%) (**a** and **c**) and *q* (mmolg^−1^) (**b** and **d**) using Visual Minteq. 2.32 code^44^ with databases feo_dlm.mdb *vs*. Visual Minteq3 code^50^ with databases feo_dlm_2008.vdb.

For Mo, the simulation results from the two Visual Minteq versions and their associated databases are similar and consistent with the experimental data. However, it has to be noted that this agreement, in E (Fig. 4a) as well as *q* (Fig. 4b) is only valid when the code default value for the FHY surface area of 600 m^2^ g^−1^ was used.

Comparing the entries in the databases for Mo surface species (Table S3), shows that the =FeOHMoO_4_^2−^ species is present in both databases (feo_dlm.mdb and feo_dlm_2008.vdb) and that the FeOMo(OH)_5_ species (logK = 17.96) from the “feo_dlm.mdb” database (used for Visual Minteq code) was replaced by the = FeMoO_4_^−^ species (logK = 9.5) in the “M4_feo_dlm.vdb” database (used for MINTEQA2 code). This replacement was done based on computational optimization but it does not seem to follow stoichiometry in terms of protonation/deprotonation (i.e., H_2_MoO_4_<=>H^+^ + HMoO_4_^−^, p*K*_a1_ and HMoO_4_^−^<=>H^+^ + MoO_4_^−2^, p*K*_a2_). In a previous study, Gustafsson^33^ simulated Mo adsorption onto FHY using the DLM and CD-MUSIC models uisng the DLM code parameters from Dzombak and Morel^30^: a specific SA of 600 m^2^ g^−1^, a fixed site density of 0.205 mol sites mol^−1^ Fe and the logK of the surface complexation reactions defining the formation of the protonated FeOH_2_^+^ species and the deprotonated FeO^−^ species were set at 7.29 and −8.93, respectively. Gustafsson^33^ concluded that in order to better describe the experimental results, beside replacing the FeOMoO_4_^−^ (logK = 9.5) with the FeOHMoO_4_^−2^ (logK = 3.14) species in the databases, a new species, defined as FeOMo(OH)_5_ (logK = 17.96) was necessary to improve the fits. The initial logK of FeOHMoO_4_^−2^ of 2.4, estimated from linear free-energy relationships, was recalculated in FITEQL resulting in a new value of 3.14. In addition, Gustafsson^33^ suggested that besides the two existing monodentate surface complexes in the DLM model, another surface complex, i.e., a bidentate complex Fe_2_O_2_XO_2_, could be important. Gustafsson and Tiberg^15^ also modelled Mo adsorption using the triple plane CD-MUSIC model but using different parameters for FHY (i.e., SA = 650 m^2^ g^−1^, PZC = 8.1, site densities of 6.25 sites/nm^2^ and 1.55 sites/nm^2^, inner and outer Stern layer capacities of 1.15 Fm^−2^ and 0.9 Fm^−2^)^15^. They found that it was necessary to include an additional outer sphere complex (i.e., FeOH_2_^½+^∙∙∙∙MoO_4_^2−^ with a logK = 11.06, calculated using PEST^59^, to complement the existing (Fe_2_O_2_MoO_2_^−^ with a logK = 18.71). Only this way could they fit well the experimental results.

In contrary, when we modelled V adsorption with VM 2.32^44^ using the feo_dlm.mdb database (Fig. 4c and d), the predicted removal efficiency and uptake capacity were ~50% lower than what our experimental results. A similar trend was observed from the simulation with the updated version (VM 3), using the feo_dlm_2008.vdb database^50^ (Fig. 4c and d), clearly showing that the used databases lack crucial solution and surface V species. If standard vanadate deprotonation pathways are considered (H_3_VO_4_<=>H^+^ + H_2_VO_4_^−^, p*K*_a1_; H_2_VO_4_^−^<=>H^+^ + HVO_4_^−2^, p*K*_a2_; HVO_4_^−2^<=>H^+^ + VO_4_^−3^, p*K*_a3_) dissociation constants for these three species should be present but: (i) the database comp_2008.vdb contains only mono-protonate vanadate species, HVO_4_^−2^; (ii) the database, thermo.vdb, only vanadate VO_4_^−3^ species (with logK = −13.49) and; (iii) the feo_dlm.mdb database (used with the VM 2.32 code^44^) contains four vanadate surface associated species, with the corresponding logKs, but only three of these follow the stoichiometric vanadate dissociation stages (Table S4). In contrast, the database linked to the newer version of the VM software (VM 3) contains only one V species, FehOHVO_4_^−3^ with a logK of −0.73 (Table S4), and values for the V species FehOHVO_4_^−3^: vary between −0.73 and −16.63 in the different databases. This comparison reveals that modelling of Mo and V sorption onto FHY is currently still difficult because the appropriate species and their dissociation constants need to be first experimentally determined. Furthermore, caution and cross-checking all existing constants prior to performing simulations has always to be done because as we show the results can differ substantially.

### Effect of surface area and particle size

**Figure 5:**
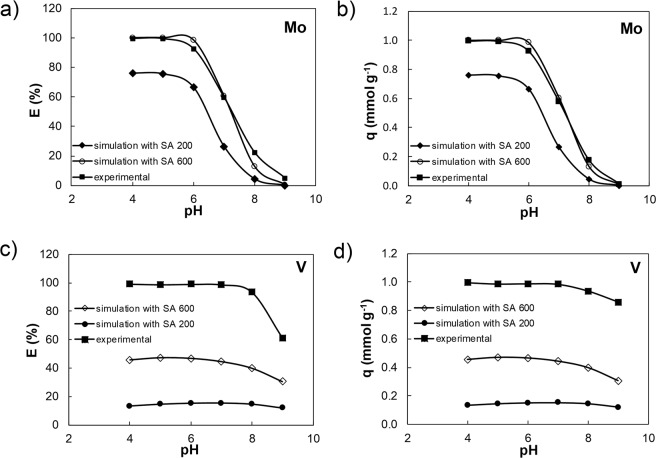
Comparison of the Mo (**a** and **b**) and V (**c** and **d**) experimental and simulation adsorption results (using Visual Minteq3 code^50^ with feo_dlm_2008.vdb database). Data are expressed as E (%) (**a** and **c**) and *q* (mmol g^−1^) (**b** and **d**), using an experimentally determined surface area of 200 m^2^ g^−1^, compared to the code’s default value of 600 m^2^ g^−1^.

As mentioned above surface area for FHY can vary dramatically (between 120 and 840 m^2^ g^−1^). We tested how using our measured SA (i.e., 200 m^2^ g^−1^) changes the adsorption outputs compared to the default value of 600 m^2^ g^−1^. Although our SA value was a third of the default SA, a good match for the Mo between the two SA values was achieved, both in terms of E (%) and *q* (mmolg^−1^), yet they were both ~1/3 higher than the experimental results (Fig. 5a and b).

For V, our measured lower SA model path plotted ~1/3 lower than the default high SA path, yet both did not fit the experimental values (Fig. 5c and d). This discrepancy could also likely be due to the SA measuring protocol, which includes a freeze-drying pre-treatment of the slurry. The existing SA measurement protocol has used dry solid as opposed to hydrated slurry that is dispersed in solution during the sorption. Thus, it is better to develop new high-resolution techniques that have capability to measure SA in solution.

In addition, the removal efficiency (E, %) trend at pH above 7, decreased smoothly in the simulations, yet sharply in the experimental results. This discrepancy is likely a consequence of the lack of V species within databases at pH > 7–8.

Finally, we tested the effect of particle size variations that we measured by DLS and TEM (see methods and SI for further information) on the adsorption capacity. Using values that varied between individual particles of 4 nm in diameter and all the way to aggregate sizes of 75 µm (Fig. S5) revealed no change in adsorption profiles, indicating that apparently particle sizes does not seem to have an effect (which is theoretically invalid) or that the model calculations are not sensitive enough for variations in particle size.

### Experimental and modelling cross-correlations from EXAFS measurements

**Figure 6:**
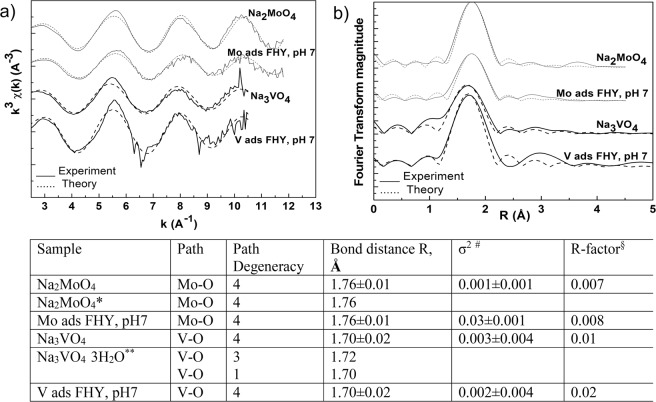
Molybdenum and Vanadium k^3^ χ(k) weighted K edge EXAFS spectra (**a**) and phase-corrected Fourier-transform (**b**) of EXAFS spectra for the experimental samples together with the Na_2_MoO_4_ and Na_3_VO_4_ standards (*Na_2_MoO_4_ structure (ICSD 4156) after Matsumoto *et al*.^64^; **Na_3_VO_4_·3H_2_O structure (ICSD 62533) after Kato and Takayama-Muromachi^65^). Below is a table with derived data with σ^2 #^representing the mean-square disorder in the distribution of interatomic distances^62^ and R-factor^§^ the sum of the residual^62^.

We confirmed these results by high resolution X-ray adsorption spectroscopy (XAS), which gave us a direct measure of Mo and V bonding onto FHY surface at pH 7. Our XAS measurements (Fig. 6a) revealed matching amplitudes, shapes and positions to the used molybdate and vanadate standards in the Mo- and V- k^3^ χ (k) weighted K edge EXAFS spectra of Mo and V adsorbed onto FHY show. Moreover, the phase corrected Fourier transformed spectra (Fig. 6b) showed four coordinated oxygen atoms at distances of 1.76 Å for Mo and 1.70 Å for V, indicating tetrahedral coordinated molybdate and vanadate species adsorbed onto FHY. An additional peak at a distance around 2.8 Å for V and 3.7 Å for Mo, could suggest a scattering signal from potential neighbouring iron surface sites. Fitting a second shell was constrained by the quality of the collected data (i.e., the k space range, especially for V; 3–10 A^−1^). In addition, it proved difficult to find Mo/V/Fe containing minerals/compounds with similar structures (e.g., corner or edge sharing of Mo/V tetrahedra bonded to Fe octahedra), needed to generate appropriate paths. Unfortunately, the previous studies also lack this piece of information. However, we tested various possibilities and these are explained in more detail in the SI.

A comparison with literature data proved difficult because only few XAS studies on Mo adsorption onto FHY at similar conditions exist. Most available data are from experiments at higher IS and at different Mo and FHY concentrations^5,13,15,33^. For example, at an IS of 0.01 and a pH of 7.3, Gustafsson and Tiberg^15^ showed that Mo forms an edge sharing bidentate complex^15^, which was similar to the findings of Kasiwabara *et al*.^5,13^ at pH 6 and 8 and IS of 0.01^5,13^.

For V adsorbed onto FHY at IS of 0.01 and pH 3.3, 3.6, 6.5 and 9.3, EXAFS spectra from Larsson *et al*.^46^ revealed that vanadate was bound primarily as an edge sharing bidentate complex with V-Fe distances around 2.8 Å^46^. For goethite, Peacock and Sherman (year 2004)^45^, showed that V adsorbs at pH 3 to 9 and high IS (0.1) as a bidentate corner sharing inner sphere complex with a possible contribution from a VO_2_(O, OH)_2_ edge sharing complex at pH 8.3.

Knowing that a decrease of IS leads to an increase of Stern layer thickness at sorbent surfaces^32^, explains the slight differences in Mo and V adsorbed surface species. At the lower IS and neutral pH represented by our data, the fingerprint interpretation of the Mo and V signals for further shells may empirically suggest contributions from tetrahedral molybdate and vanadate species forming corner and edge sharing outer sphere complexes. However, this possibility has to be confirmed by further analyses over a larger k-space interval.

## Summary

We showed that combining experimentally derived FHY characteristics for surface area (~200 m^2^ g^−1^), surface charge (PZC 7.89), particle/aggregate size (4 nm to 75 µm) with quantitative and pH dependent adsorption data resulted in V having a higher affinity for FHY surface sites compared to Mo, a difference easily explained by the V and Mo anion sizes and valences. In addition, the sorption experiments revealed a change in the sorption mechanism as a function of the pH and anion valence. The comparison with the modelling outputs using the GW^58^ and VM^50^ codes showed a reasonable agreement for Mo, but quantitative differences for V. Furthermore, the comparison between the adsorption simulation outputs and the experimental results revealed that: varying SA, using different geochemical codes and different databases, lead to rather large differences in outputs:

(i) qualitatively, the important Mo and V– FHY surface species were FeOMo(OH)_5_ and FeOHMoO_4_^−2^ for Mo and FeOHVO_4_^−3^ for V; however, this V species only partially described the experimental results;
(ii) quantitatively, for Mo the simulated pH adsorption edge (and removal efficiency) matched the experimental results only when the code default SA for FHY (600 m^2^ g^−1^) was used, for V the simulated pH-adsorption edge was ca. 50% lower than experimental results, over the entire pH range.

These discrepancies are likely a consequence of incomplete databases (i.e., presence or absence of appropriate and inappropriate species with or without accurate logK constants) as well as differences in adsorbent characteristics (*i.e*., surface area, particle geometry and size, dissociation constants, etc.).

This comparison leads us to suggest that the modelling of experimental data using geochemical speciation modelling and adsorption simulations for Mo and V currently still require more experimental data for the models to match the experimental data. Although our limited EXAFS results (only at pH 7) provides a direct assessment of the Mo and V surface species, what is needed is data over a much wider chemical space in terms of pH and ionic strengths. Combined with a rigorous check of used database entries and an quantitative assessments of possible species involved in the adsorption is needed, in order to choose the correct stoichiometric species, and to identify the sources of intrinsic constants.

Finally, our results showed that improvements in geochemical databases and models are required before more quantitative insight of the geochemical processes occurring in various media and environmental conditions related to Mo and V sorption to FHY can be made.

## Methods

### Ferrihydrite synthesis and characterization

Two-line ferrihydrite (FHY) was synthesized following the method of Cornell and Schwertmann^22^. All solutions were prepared from analytical grade from Fisher Scientific. Once synthesized, consecutive washing of the ferrihydrite slurry using MiliQ water was done until the total dissolved solid (TDS) (measured with Water PAL Sprinte™ Industries TDS meter) was very low (~10 ppm). The resulting slurry was subsequently stored in a sealed polypropylene bottle at 4  C and pH 7. In all adsorption experiments (described below) FHY was used as a slurry because drying the FHY would result in changes in its characters such as surface area, particle size and crystallinity. It is, however, noted that the results described in this work were reported as dried weight for FHY. The conversion from wet slurry to dried weight for FHY was done taking into account the density of the slurry which was previously determined by drying sacrificial slurry samples overnight at 40–50 °C.

For phase identification and surface area determinations, FHY was also freeze-dried (EC Modulyo freeze drier at −50 °C and 7 mbar) followed by analyses. Its surface area (SA) was determined by the Brunauer, Emmett and Teller method (BET, Micromeritics Gemini V, He gas, evacuation rate of 100 mmHg min^−1^, 765 mmHg, 5 seconds equilibration time) while for phase identification we used X-Ray Diffraction (XRD, Bruker D8, angle 5–70°, step size 0.01, power 40 kV and 30 mA). The FHY particle size distribution in solution at variable IS (0, 0.01, 0.7 and 1) was measured by dynamic light scattering (DLS, Malvern Mastersizer). In addition, the FHY surface charge properties were measured using potentiometric titrations of freshly synthetized FHY and using a Man-Tech Inc. auto-titration system equipped with an automatic burette (PC-TitrateTM) and three ports: a precise glass pH electrode, a high-resolution titrant injector and a thermometer.

Synchrotron-based X-ray absorption spectroscopy (XAS) was used to investigate the valence state and bonding environment (interatomic distances, coordination number, and species of the neighbours of the absorbing atom) of Mo and V associated with FHY after adsorption. XAS measurements were carried out at station 16.5 at the Daresbury Laboratory, (the Synchrotron Radiation Source, SRS)^60^ for Mo, and at the I18-Microfocus spectroscopy beamline (the Diamond Light Source, DLS)^61^ for V. The I18-Microfocus spectroscopy station set-up was as described in Brinza *et al*.^53^. Slurry samples from the adsorption experiments were examined in fluorescence mode, while standards (Na_2_MoO_4_, K_2_MoO_4_ and MoO_3_ for Mo and Na_3_VO_4_ and V_2_O_5_ for V, diluted with boron nitride) were investigated in transmission mode. Na_2_MoO_4_ and Na_3_VO_4_ were used as guidance to generate modelling (single and multiple scattering) paths for fitting the experimental data in the range of 2–10 for k^3^-weighted EXAFS and 1–3.5 for Fourier Transform (FT). Data normalization and analysis were done using the Athena and Artemis software - both part of the Demeter package^62^. The goodness of EXAFS data fitting is given by R factor, which represents the absolute misfit between the theory and the data^62^.

### Adsorption experiments

Adsorption studies were carried out in batch mode (in 500 mL beakers) with FHY slurries (equivalent to 0.1 gL^−1^) equilibrated with a 100 µM metal-containing solution (Mo or V) at room temperature (25 °C ± 2) and under continuous stirring (300 rpm). All experiments were conducted under N_2_ atmosphere to minimise CO_2_ in the system. Slurry aliquots were taken at a specific times (0; 10; 20; 40; 80; 160; 320 and 1280 minutes) to be able to derive and then model the Mo and V adsorption kinetics^54,63^). Solution samples were separated by filtration from the solids using 0.4 µm syringe filter and then analysed using inductively coupled plasma optical emission spectrometry (ICP-OES, Perkin Elmer Optima ICP-OES; detection limit Mo 0.6 µg L^−1^ and V 0.9 µg L^−1^). The effect of pH was studied across the pH range between 4 and 9. All adsorption experiments were carried out using a potentiometric titrator (Radiometer 865 from Titra-Lab^®^), by adding acid (0.1 N HCl) or base (0.1 N NaOH) at a flow rate of max. 1 mL min^−1^. Acid and base volumes added for each experiment at a specific pH were monitored and collected by the TitraMaster 85 (V4.0) software. The experiments were carried out at an ionic strength value of max 0.0012 for V and 0.0005 for Mo systems.

The experimental results of the pH dependent adsorption studies were plotted as *q* against pH and E against pH, where *q* is the metal uptake capacity (mmol g^−1^) and E is the uptake efficiency in % (the formulas are detailed in the SI).

The adsorption kinetics (expressed as *q* against time (and E against time, see SI)) were than modelled using both a pseudo-first order kinetic model (PFO) and pseudo-second order kinetic model (PSO)^54–56^ to calculate kinetic rates and weighed uptake capacity values and to derive adsorption mechanisms.

### Geochemical modelling and adsorption simulation

It is important to note that the term”modelling” was used for the speciation calculation of elements in solution and the term”simulation” was used for the adsorption modelling.

#### Geochemist’s Workbench^®^ 6.0

The speciation modelling using Geochemist’s Workbench^®^ 6.0 code^58^ aimed to identify the aqueous or solid species present in the adsorption systems as well as the saturation limits for Mo and V compounds. The speciation modelling is also to determine the optimal conditions and the metal speciation as a function of pH in the experimental adsorption studies. Speciation diagrams as a function of pH were calculated using the thermodynamic data “thermo2000.dat” for Mo (VI) and “thermo_minteq.dat” for V (V) at standard conditions (i.e., T = 25 °C, P = 1.013 bar).

#### Visual Minteq (VM)

Adsorption simulations of the experimental adsorption studies were done with the geochemical software Visual Minteq (VM) version 2.32^44^ and version 3.0^50^ using the experimental adsorption conditions (e.g., pH, IS, metal and sorbent concentrations, temperature, pressure, CO_2_ free systems, measured and empirically derived SA and particles size (PS) properties, etc.). VM can be used to compute equilibrium among dissolved, adsorbed, solid, and gas phases in a system at specified conditions. It is based on the MINTEQA2 version 4.0 code which was compiled in Visual Basic 6.0 in 2005^44^ and updated in 2008 and 2012. The code includes extensive databases such as: thermodynamic database (i.e., thermo.mdb), components database (i.e., comp.mdb), solids database (i.e., type6.mdb), Dissolved Organic Matter (DOM) complex database (gaussian.mdb) and complexation database (feo-dlm.mdb), but also options for surface complexation models (*e.g*., 2 p*K*_a_- DLM, 2p*K*_a_-TLM, 2p*K*_a_-CCM, HFO-DLM and more recently Fh-2site, Fh-3site). Adsorption data were modelled with the Hydrous Ferric Oxide (HFO) model, and a diffuse layer model (DLM) with 2pK. The HFO model contains as default SA of 600 m^2^ g^−1^ (in the 2.30 version SA is fixed at the default value, whereas the version 3.0 allows changes in SA); two type of sites with densities of 2.25 sites/nm^2^ and 0.056 sites/nm^2^; FHY surface sites concentration 0.07496 mmol L^−1^ and 0.00187 mmol L^−1^; FHY molecular weight 106.86 gmol^−1^; the FHY atoms stoichiometry: of Fe^+3^:H_2_O:H^+^ as 1:3:-3*; with log K’s of the surface complexation reactions defining the formation of the protonated FeOH_2_^+^ and deprotonated FeO^−^ species as 7.29 and −8.93, respectively. In addition, it contains optional particle geometry choices from: plane, cylinder and sphere, and adjustable particle sizes. In our model one shape with two sizes was selected (Table 2; additional discussion in SI). Adsorption simulations were done using the following databases: thermo.mdb (thermo.vdb in VM 3.0 version), type6.mdb type6.vdb in VM 3.0 version), comp.mdb (comp_2008.vdb in VM 3.0 version) and gausian.mdb (gaussian.vdb in VM 3.0 version) and two complexation databases: feo dlm.mdb (feo_dlm_2008.vdb in VM 3.0 version) as available with VM codes^44,50^. A third complexation database, M4_feo_dlm.vdb, was also discussed in SI.

**Table 2:** Parameters used in the VM adsorption simulation.

Parameter	Value	Reference
Ionic strength	0.001	This study
Mo(VI) or V(V) concentration	100 µM	This study
Ferrihydrite surface area (measured)	200 m^2^ g^−1#^	This study
Ferrihydrite particles concentration (Cp)	0.1 g L^−1^or 0.935 mM^±^	This study
Radius of a considered spherical particle	4 nm and 75000 nm*	This study
logK for = FeOMo(OH)_5_	17.96	Feo_dlm_2008
logK for = FehOMo(OH)_5_		
logK for = FeOHMoO_4_^−2^	3.14	Feo_dlm_2008
logK for = FehOHMoO_4_^−2^		
logK for = FeOHVO_4_^−3^	−0.73	Feo_dlm_2008
logK for = FehOHVO_4_^−3^		

^±^Cp is calculated as 0.935 mM (assuming a ferrihydrite stoichiometry of Fe:H_2_OH:H as 1:3:-3, with FHY molecular weight of 106.86 g mol^−1^).

^#^Measured surface area (200 m^2^ g^−1^) was also used in the adsorption simulation in order to compare the simulation results with the experimental data. A value of 600 m^2^ g^−1^ for the surface area is the default for the HFO model.

*Particle shape and size chosen in the simulation: spheres with sizes between 4 nm (measured by TEM^53^ and 75000 nm (measured in solution, this study).

For the simulated uptake capacities, the amount of Mo and V taken up by FHY was solely quantified from the concentrations of the surface associated species (in mmols), which were summed and normalized to 1 g of FHY. The adsorption simulation outputs using the VM code^44,50^ (varying code version with associated databases and FHY parameters SA (and PS, in SI)) were compared with the experimental results and discussed. With this study we aimed only to compare our new experimental data on Mo and V adsorption with existing databases and model outputs and not to change modelling codes or databases.

## Supplementary information

Experimental and simulation results of the adsorption of Mo and V onto ferrihydrite
