# Supplementary material for: Experimental and simulation results of the adsorption of Mo and V onto ferrihydrite
Source: Sci Rep. 2019 Feb 4;9:1365. doi: 10.1038/s41598-018-37875-y (PMC6362054; doi:10.1038/s41598-018-37875-y)
Supplement: Supplementary file 1 — Experimental and simulation results of the adsorption of Mo and V onto ferrihydrite [file 41598_2018_37875_MOESM1_ESM.docx]

**Experimental and simulation results of the adsorption of Mo and V onto ferrihydrite**

**Loredana Brinza*^, a, b^, Hong Phuc Vu^b, c^, Mariana Neamtu^a^_,_ Liane G Benning^b, d, e^**

*^a^ Alexandru Ioan Cuza University, Department of Interdisciplinary Research, Iasi 700107, Romania*

*^b^ University of Leeds, School of Earth & Environment, Earth Surface Sciences Inst, Leeds LS2 9JT, West Yorkshire, United Kingdom*

*^c^ University of Melbourne, The Faculty of Science, School of Earth Sciences, Melbourne, Victoria 3010, Australia*

*^d^ GFZ German Research Centre for Geosciences, D-14473 Potsdam, Germany*

*^e^ Department of Earth Sciences, Free University of Berlin, 12249* *Berlin, Germany*

**Supplementary information**

**------------------------------------------**

* Correspondence to: E-mail address: [b_loredana@yahoo.com](mailto:b_loredana@yahoo.com), [loredana.brinza@uaic.ro](mailto:loredana.brinza@uaic.ro)

**Uptake capacity *vs.* removal efficiency:**

The experimental and modelling results are expressed as uptake capacity (or so called surface loading) and as removal efficiency (the degree of element removed from the solution – measured from metal concentration in supernatant):

$q=\frac{V\cdot\left(C_{i}-C_{t}\right)}{S_{a}}$ (Eq. 1)

where: *q* is the metal uptake capacity, mg g^-1^; V is the solution volume, L; C_i_ is the initial metal concentration in solution, mg L^-1^; C_t_ is the metal concentration in solution at time t, mg L^-1^; S_a_ is the dry weight of FHY per adsorption batch, g;

$E=\frac{C_{i}-C_{t}}{C_{i}-C_{f}}\cdot100$ (Eq. 2)

C_i_ is the initial metal concentration in solution, mg L^-1^; C_t_ is the metal concentration in solution at time t, mg L^-1^; C_f_ is the final metal concentration in solution, mg L^-1^.

These two expressions offer two distinct views of adsorption data: with adsorbent properties included (*q*) and without (E). For the simulated uptake capacities, the amount of Mo and V taken up by FHY was quantified only from the concentrations of the surface associated species (in mmols), which were summed and normalized to 1 g of FHY.

**Kinetic models**

A pseudo-first order (PFO) kinetic model^1^ and a pseudo-second order (PSO) kinetic model^2^ were used to fit the experimental data^3^ up to equilibrium and including equilibrium data, following a similar approach as described by Simonin 2016^4^.

The PFO kinetic model is given by Eq. S3:

$\frac{dq}{dt}=k_{1}(q_{e}-q_{t})$ (Eq. S3)

where: q_t_ and q_e_ are the amount of solute sorbed per mass of sorbent (mmol g^−1^) at any time (t) and equilibrium (e), respectively, and k_1_ is the rate constant of first-order sorption (min^−1^).

The PSO kinetic model is represented by Eq. S4

$\frac{dq}{dt}=k_{2}{(q_{e}-q_{t})}^{2}$ (Eq. S4)

where: k_2_ is the rate constant, (g mmol^−1^ min^−1^); q_t_ is the metal uptake capacity (mmol g^-1^) at any time t (min).

The choice of which model better fits the experimental data was based on comparing statistical parameters such as R^2^, Adj. R^2^, average absolute deviation (AAD) and the residuals of the fit. R^2^ and Adj. R^2^ parameters were needed to differentiate between the best fits of scenarios in which different and all numbers of data points were fitted. R^2^ allows comparison between PFO and PSO kinetic models with different numbers of data points – i.e., time when equilibrium was reached but not including the equilibrium platter (varied function of pH). Whereas Adj.R^2^ allows comparison between the models when the same number of data points were considered – i.e., data including equilibrium. If the above parameters were very similar, then the minimal value of AAD and the uniformity of residual profile were used to decide the difference between the best fit models. Plots of the data fitted up to equilibrium and the obtained associated parameters are presented in the main paper whereas the results from fitting all the data points at equilibrium are displayed for comparison below.

**Ferrihydrite particles/cluster size in solution: dynamic light scattering results**

FHY particle size distribution in solution was measured at different ionic strengths (0, 0.01, 0.7 and 1) under dynamic regime by Dynamic Light Scattering (DLS) (a Malvern Mastersizer instrument with the following settings: particle refractive index, 2.98; particle absorption index, 1; dispersant name, water; dispersant refractive index, 1.33) The Mastersizer 2000 software contains the Mie scattering model allowing to accurately measure the particle size (from 0.02 μm to 2000 μm) across a wide dynamic range (instrument called MalvernLab 2000).

**Figure S1**. Particle size distribution at different ionic strengths and same particle concentration (0.002 g L^-1^). Average of 20 measurements from Malvern Mastersizer 2000 (laser diffraction).

Measurements of particle size in solution using DLS (Fig. S1.) revealed that FHY in solution is not monodispersed but forms clusters with size distribution varying function of the solution’s ionic strength. Thus, at very low ionic strengths FHY in solution in dynamic regime (under stirring conditions) occurs as clusters with 3-150 µm in size, averaging 75 µm. This value was chosen to be used in the adsorption simulation test (see later).

**Table S1.** Summary of the results from potentiometric titration: point of zero charge of the FHY at different ionic strengths and appropriated pKa value

| IS electrolyte | Vol NaOH (mL) | End point | pH p*K*_a_ |
| --- | --- | --- | --- |
| 0.01 | 15.65 | 7.96 | 6.23 |
| 0.1 | 2.05 |  |  |
| 1 | 0.55 |  |  |

All titrations were carried out using a Man-Tech Inc. auto-titration system equipped with an automatic burette (PC-TitrateTM) and three ports: a glass pH electrode, a titrant injector and a thermometer. Titration was conducted in 250 mL beakers on a magnetic stirrer plate (600 rpm). Prior to each titration, the pH electrode was manually calibrated using a VWR certified pH standard solution. The working temperature was room temperature (21 ± 0.5 °C). A standard titration protocol involved equilibrating a FHY suspension in 100 mL of electrolyte background solution (NaCl, 0.01, 0.1 and 1 N), then titrating both from high to low pH and from low to high pH with either HCl or NaOH (0.01, 0.1 and 1 N) to establish hysteresis effects.

**Adsorption kinetic – indirect approach for understanding sorption mechanisms**

***Kinetic profiles of Mo and V removal efficiency from solution***

**Figure S2.** The kinetic profiles of molybdenum (a) and vanadium (b) removal efficiency by FHY (C_i_=100 µM metal and C_FHY_ =0.1 g L^-1^) at different pH values in the mono-sorbate systems; symbols represent experimental data and lines show the fitting to a pseudo-second order kinetic model. The error bars corresponding with metal concentrations are not visible due to the size of the symbols.

The Mo and V removal efficiency values (Fig. S2.a and b) indicate that the adsorption/removal efficiency for Mo is high at pH below 6 (90-100%), but decreased with increasing pH (i.e., pH 7 < 60%, pH 8 < 20% and pH 9 < 3%), while the adsorption efficiency for V decreased to ~ 60% only at pH 9. This pH dependent behaviour revealed that below pH 6, both V and Mo have the highest affinity for the FHY binding sites (Fig. S2.a and b). Ferrihydrite loading in the Mo and V adsorption studies is expressed as uptake capacity (q) and plotted in Fig. 1, main text. For both Mo and V, the uptake capacity and removal efficiency follow the same trend: they decrease with increase in pH, but there is a larger decrease for the Mo system compared to the V system.

***Summary of the pseudo-first order and pseudo-second order kinetic model parameters, fitted for Mo and V including data at equilibrium, presented below:***

**Figure S3.** The pseudo-first order and the pseudo-second order kinetic models fitted for Mo (a) and V (b) including all data at equilibrium.

**Table S2.** Summary of the pseudo-first order and pseudo-second order kinetic model parameters fitted for Mo and V including all data at equilibrium

| **PFO** | | | | | | | | | | **PSO** | | | | | | | | | **N***  **Max/**  **t, min** |
| --- | --- | --- | --- | --- | --- | --- | --- | --- | --- | --- | --- | --- | --- | --- | --- | --- | --- | --- | --- |
| **Mo/**  **pH** | | **Q (mmol g^-1^)** | | **k1**  **(min^-1^)** | | **R^2^** | **Adj R^2^** | **AAD** | | **Q (mmol g^-1^)** | **k2 (g mmol^−1^ min^−1^)** | | **R^2^** | | **Adj. R^2^** | | **AAD** | |  |
| 4 | | 0.993  ±0.0008 | | 0.553  ±0.003 | | **0.9999** | **0.9999** | 0.003 | | 1.018  ±0.014 | 1.317  ±0.225 | | 0.983 | | 0.982 | | 0.006 | | 13*  1280 |
| 5 | | 0.988  ±0.0016 | | 0.349  ±0.003 | | **0.9997** | **0.9997** | 0.005 | | 1.020  ±0.017 | 0.673  ±0.099 | | 0.978 | | 0.976 | | 0.009 | | 13*  1280 |
| 6 | | 0.879  ±0.017 | | 0.134  ±0.018 | | 0.982 | 0.9794 | 0.011 | | 0.909  ±0.007 | 0.304  ±0.025 | | **0.997** | | **0.997** | | 0.016 | | 9*  1280 |
| 7 | | 0.557  ±0.017 | | 0.060  ±0.0086 | | 0.968 | 0.963 | 0.013 | | 0.592  ±0.017 | 0.151  ±0.029 | | **0.976** | | **0.973** | | 0.023 | | 9*  1300 |
| 8 | | 0.214  ±0 011 | | 48.440 | | 0.867 | 0.848 | 0.0004 | | 0.214  ±0.013 | 1.3*  10^-28^ | | 0.867 | | 0.848 | | 0.0004 | | 9*  1340 |
| 9 | | 0.031  ±0.006 | | 53.037 | | 0.376 | 0.251 | 0.0009 | | 0.031  ±0.007 | 1.9*  10-^46^ | | 0.376 | | 0.251 | | 0.0009 | | 7*  1290 |
| **PFO** | | | | | | | | | **PSO** | | | | | | | | | | **N***  **Max/**  **t, min** |
| **V/**  **pH** | **Q (mmol g^-1^)** | | **k1**  **(min^-1^)** | | **R^2^** | | **Adj R^2^** | **AAD** | **Q (mmol g^-1^)** | | | **k2 (g mmol^−1^ min^−1^)** | | **R^2^** | | **Adj. R^2^** | | **AAD** |  |
| 4 | 0.989  ±0.0005 | | 0.608  ±0.003 | | **0.9999** | | **0.9999** | 0.003 | 1.012  ±0.013 | | | 1.546  ±0.273 | | 0.985 | | 0.984 | | 0.005 | 13*  1280 |
| 5 | 0.971  ±0.004 | | 0.374  ±0.01 | | **0.998** | | **0.997** | 0.005 | 1.004  ±0.014 | | | 0.737  ±0.091 | | 0.975 | | 0.984 | | 0.009 | 13*  1280 |
| 6 | 0.984  ±0.001 | | 0.438  ±0.003 | | **0.9998** | | **0.9998** | 0.004 | 1.0157  ±0.015 | | | 0.884  ±0.135 | | 0.982 | | 0.980 | | 0.008 | 13*  1280 |
| 7 | 0.970  ±0.003 | | 0.341  ±0.006 | | **0.9988** | | **0.9987** | 0.005 | 1.003  ±0.015 | | | 0.656  ±0.085 | | 0.983 | | 0.981 | | 0.010 | 13*  1280 |
| 8 | 0.9148  ±0 007 | | 0.250  ±0.009 | | **0.9954** | | **0.9950** | 0.006 | 0.951  ±0.013 | | | 0.456  ±0.042 | | 0.989 | | 0.988 | | 0.012 | 13*  1280 |
| 9 | 0.584  ±0.010 | | 0.303  ±0.028 | | 0.972 | | 0.969 | 0.003 | 0.609  ±0.007 | | | 0.874  ±0.075 | | **0.991** | | **0.990** | | 0.007 | 13*  1280 |

*N – number of data points considered, representing various time frames of adsorption progress

The PFO and PSO kinetic model parameters, fitted for Mo and V including data at equilibrium, support the results obtained from fitting the data up to equilibrium (see Table 2 in the main paper). This indicates that, for these datasets, there was not much difference whether partial or all data points were considered in the fitting. This confirmed, once again, that at pH below 5 for Mo and below 8 for V, the experimental data were well described by the PFO kinetic model and above these values the PSO model gave better fits (exception is made for Mo at pH 8 and 9, for which none of the models have fitted the data well – details in the main paper). Changes in the kinetic models best fitting the data with varying pH values support changes in mechanism as discussed in the main paper.

**Geochemical modelling: Mo and V speciation *in solution* using Geochemist Workbench**^®^ **6.0 (GW) code *vs.* Visual Minteq (VM) code**

According to the GW code and appropriate databases (see methods section), Mo in aqueous systems can form soluble anionic species, as well as precipitates, as function of concentration. Thus, at pH below 4, the Mo species are: MoO_3_ (H_2_O)_3_ (aq) – at concentrations below 10 µM and H_2_MoO_4_ (s) – at concentrations above 10 µM; whereas above this pH, MoO_4_^2-^ becomes the major species (Fig. SI 4.c). V forms soluble cationic species (i.e., VO_2_^+^) below pH 3.5 and anionic species such as: H_2_VO_4_^-^, between pH 3.5 and 8.7 and HVO_4_^2-^ above pH 8.7, as major species (Fig. S4.d). All the above speciation occurs at V concentration in solution below 1 mM. Above this concentration under acidic environment, V can polymerize and even precipitate as V_2_O_5_ solid species (Fig. S4.d).

**Figure S4.** Mo (a, c) and V (b, d) speciation as a function of pH and concentration (as activity) by Geochemist Workbench^®^, old database (a and b) and new database (c and d). The blue zone indicates aqueous phases whereas the yellow zone indicates solid phases. The diagram was created using the Geochemist Workbench^®^ 6.0 code following standard conditions (pressure, 1 atm; temperature, 25 °C; IS 0.001). The blue lines indicate Mo/V concentrations used in adsorption experiments. Mo (e) and V (f) speciation at different pH values under standard conditions (pressure, 1 atm; temperature, 25 °C; in distilled water and metal concentration of 100 μM) by Visual Minteq.

According to VM^5^, which can display species at our working concentration (100 µM), 100% of Mo is present as MoO_4_^2-^ at pH 6 to 10. At pH 5 only 85% of Mo is molybdate beside 13% HMoO_4_^-^ and 2% MoO_3_(H_2_O)_3_ (molybdenum trioxide tri-hydrated) (Fig. S4.e). Conversely, H_2_VO_4_^-^ is the most common species (80-97%) at pH between 4 and 8 and HVO_4_^2-^ becomes the dominant species at pH above 8. About < 5-10% of other V species (*e. g.,* VO_4_^3-^, HV_2_O_7_^3-^, VO^2+^, etc.) are also present in solution at most pH values (Fig. SI4.f).

Comparing Mo and V speciation modelling outputs from the GW code^6^ (Fig. S4.c and d) and VM code^5,7^ (Fig. S4.e and f), the results differ slightly, qualitatively and quantitatively:

- For Mo, according to the GW code^6^ (which used “thermo2000.dat” database), Mo species are: MoO_3_(H_2_O)_3_ and H_2_MoO_4_ at pH below 4 and MoO_4_^2-^ above it; whereas, according to the VM code ^5^ (which used ”thermo.mdb” database), MoO_4_^2-^ (85%), HMoO_4_^-^ (13%) and MoO_3_(H_2_O)_3_ (2%) are present at pH 5 and MoO_4_^2-^ is the sole species at pH above 6. Furthermore, qualitatively, it can be noted that, at pH 4 and Mo concentration of 100 µM (= log a MoO_4_^2-^=-4), for example, the H_2_MoO_4_ species appears as solid phase (precipitate) in GW^6^, but is not present in the VM code^5^ (instead, MoO_3_(H_2_O)_3_ (̴ 37%), HMoO_4_^-^ (̴ 37%), and MoO_4_^2-^ (25%) are shown as aqueous species).
- For V, according to the GW code^6^ (which used “thermo_minteq.dat” database), the species are: VO2^+^ at pH below 3.5, H_2_VO_4_^-^ at pH between 3.5-8.7 and HVO_4_^2-^ above pH of 8.7; whereas, according to the VM code^5^ (which used ”thermo.mdb” database), H_2_VO_4_^-^ is the most common species (80-97%) at pH between 4 and 8 and HVO_4_^2-^ becomes the dominant species at pH above 8. In addition, VM code output shows 5-10% of other V species (*e. g.,* VO_4_^3-^, HV_2_O_7_^3-^, VO^2+^) as being present in solution at most pH values. Qualitatively, it can be observed that at high pH, V speciation computed by both codes is similar, whereas under acidic conditions the GW code outputs the VO_2_^+^ (at pH below 3.5) as a major species (beside H_2_VO_4_^-^), whereas the VM code^5^ outputs this species as a very minor one.

These discrepancies are due to differences in code database inputs (e.g., intrinsic constants, species type). They can make an important point of view for geochemists, especially if access to all these codes is limited. It is recommended that database inputs should be checked, merged, integrated and universally formatted in order to be read and used by all available geochemical modelling codes to enable users to extract accurate information: exact species that interact with sorbents and to adsorption mechanism at macroscale.

**EXAFS results - second shell fitting discussion**

Despite the second shell fitting constraints, mentioned in the main text, for Mo efforts were made to generate Mo-Fe longer paths by using structures containing Fe octahedrons and also Mo octahedrons^8^ and tetrahedrons^9^. The results showed that there was not a realistic Fe site at a distance of about 3.6-3.7 Å, which could have confirmed the presence of corner sharing complex. Thus, the formation of a potential outer sphere complex for Mo could only be speculated.

Considering schubnelite^10^ as the sole mineral containing sharing V tetrahedrons and Fe octahedrons, a V-Fe path (at atoms distance of 3.49 Å) was generated and used in fitting. The misfit for this scenario enables the exclusion of the formation of corner sharing complexes at FHY surface. Because distances around 2.9 ± 0.2 Å are typically attributed to edge sharing vanadate tetrahedral bound to iron octahedra^11,12^, it is speculated that this is likely to be the case for V bonding at pH 7 and IS of 0.001.

**Molybdenum and vanadium adsorption simulation: *code version and database effect***

**Table S3.** Molybdenum surface associated species found in various databases

| Database name | Mo surface associated species |
| --- | --- |
| M4_feo_dlm.vdb | 16124800,**"=FehMoO4-"**,0,**9.5**,0,0,-1,0,0,0, 0,5,1,1612,1,480,1,330,-1,2,-1,1601  16114800,**"=FeMoO4-"**,0,**9.5**,0,0,-1,0,0,0, 0,5,1,1611,1,480,1,330,-1,2,-1,1601  16124801,**"=FehOHMoO4-2"**,0,**2.4**,0,0,-2,0,0,0, 0,3,1,1612,1,480,-2,1601  16114801,**"=FeOHMoO4-2"**,0,**2.4**,0,0,-2,0,0,0, 0,3,1,1611,1,480,-2,1601 |
| feo_dlm.mdb | 16124800,**"=FehOMo(OH)5"**,0,**17.96**,0,0,0,0,0,0,0,4,1,1612,1,480,2,330,1,2  16114800,**"=FeOMo(OH)5"**,0,**17.96**,0,0,0,0,0,0,0,4,1,1611,1,480,2,330,1,2  16124801,**"=FehOHMoO4-2**",0,**3.14**,0,0,-2,0,0,0,0,3,1,1612,1,480,-2,1601  16114801**,"=FeOHMoO4-2"**,0,**3.14**,0,0,-2,0,0,0,0,3,1,1611,1,480,-2,1601 |
| feo_dlm_2008.vdb | 16124800,**"=FehOMo(OH)5"**,0,**17.96**,0,0,0,0,0,0,0,4,1,1612,1,480,2,330,1,2  16114800,**"=FeOMo(OH)5"**,0,**17.96**,0,0,0,0,0,0,0,4,1,1611,1,480,2,330,1,2  16124801,**"=FehOHMoO4-2**",0,**3.14**,0,0,-2,0,0,0,0,3,1,1612,1,480,-2,1601  16114801**,"=FeOHMoO4-2"**,0,**3.14**,0,0,-2,0,0,0,0,3,1,1611,1,480,-2,1601 |

**Table S4.** Vanadium surface associated species found in various databases

| Database name | V surface associated species |
| --- | --- |
| Feo_dlm.mdb | 16129031,"**=FehH2VO4**",0,**22.4**,0,0,0,0,0,0,0,3,1,1612,1,903,2,330  16119031,"**=FeH2VO4**",0,**22.4**,0,0,0,0,0,0,0,3,1,1611,1,903,2,330  16129031,"**=FehHVO4-**",0,**16.2**,0,0,-1,0,0,0,0,4,1,1612,1,903,1,330,-1,1601  16119031,"**=FeHVO4-**",0,**16.2**,0,0,-1,0,0,0,0,4,1,1611,1,903,1,330,-1,1601  16129032,"**=FehVO4-2**",0,**9**,0,0,-2,0,0,0,0,3,1,1612,1,903,-2,1601  16119032,"=**FeVO4-2**",0,**9**,0,0,-2,0,0,0,0,3,1,1611,1,903,-2,1601  16129033,"**=FehOHVO4-3**",0,**-0.73**,0,0,-3,0,0,0,0,4,1,1612,1,903,-1,330,-3,1601  16119033,"**=FeOHVO4-3**",0,**-0.73**,0,0,-3,0,0,0, 0,4,1,1611,1,903,-1,330,-3,1601 |
| Feo_dlm_2008.vdb | 16129031,"**=FehOHVO4-3**",0,**-.73**,0,0,- 3,0,0,0,0,4,1,1612,1,903,-1,330,-3,1601  16119031,"**=FeOHVO4-3**",0,**-.73**,0,0,-3,0,0,0,0,4,1,1611,1,903,-1,330,-3,1601 |

**Mo and V adsorption onto FHY - particles/clusters size effect (TEM observed (i.e., 4 nm) *vs.* measured in solution (75000 nm))**

Among FHY parameters that are important in the adsorption process, beside SA, particle size (PS) is another parameter discussed. Our previous work on FHY characterization by TEM showed that FHY individual particles synthetized with this method have a size of 3-5 nm^13^. The DLS results indicated that FHY in solution forms aggregates with average size of 75 µm. Thus, in this respect, in our modelling we have chosen to compare experimental results with simulation outputs in which we used FHY particle spheres with a size of 4 nm *vs.* 75 µm (see below).

Figure S5 shows VM^7^ simulation of particle size effect on Mo and V sorption pH profile *vs.* experimental results. In this set, we assumed that the correct SA is 600 m^2^g^-1^, considering the feo_dlm_2008 database, and particle geometry as sphere with PS set to 4 *vs.* 75000 nm. The 4 nm value was chosen from our previous TEM measurements^13^ and the 75000 nm value was chosen as the average value given by our DLS experimental measurements of FHY in solution at very low ionic strength (see DLS results above).

**Figure S5.** Comparison of the Mo (a and b) and V (c and d) experimental and simulation adsorption profiles (using Visual Minteq 3 code^7^ with databases feo_dlm_2008.vdb, code default SA as 600 m^2^g^-1^) expressed as E (%) (a and c) and q (mmol g^-1^) (b and d), using the experimentally determined cluster size of 75000 nm in solution *vs.* TEM observed particles of 4 nm.

For Mo (Fig. S5.a and b) and V (Fig. S5.c and d) the simulation results showed no and little differences, respectively, between the 4 and 75000 nm particle/cluster size, in both E (Fig. S5.b and d) and q expression modes (Fig. S5.a and c). Similar outputs were obtained when SA of 600 m^2^g^-1^ or 200 m^2^g^-1^ was used for both PS simulations (data not shown). This indicates that although the code contains the option of changing this variable, it does not compute a sensible and realistic output. This could be due to a coding/scripting deficiency in the model, such as a computation error or the lack of a formula that correlates sphere geometry and size with sorbent surface properties. Further investigation and appropriate improvements are required, from a user point of view. The expected trend was as follows: an increase of PS would lead to a decrease of SA (and also a decrease of sorption uptake capacity) and vice versa.

**References**

1 Lagergren, S. Zur theorie der sogenannten adsorption gelöster stoffe,  K*ungliga Svenska Vetenskapsakademiens*. *Handlingar* **24**, 1-39 (1886).

2 Ho, Y. S. & McKay, G. A Comparison of Chemisorption Kinetic Models Applied to Pollutant Removal on Various Sorbents. *Process Safety and Environmental Protection* **76**, 332-340, doi:<https://doi.org/10.1205/095758298529696> (1998).

3 Plazinski, W., Rudzinski, W. & Plazinska, A. Theoretical models of sorption kinetics including a surface reaction mechanism: A review. *Advances in Colloid and Interface Science* **152**, 2-13, doi:<https://doi.org/10.1016/j.cis.2009.07.009> (2009).

4 Simonin, J.-P. On the comparison of pseudo-first order and pseudo-second order rate laws in the modeling of adsorption kinetics. *Chemical Engineering Journal* **300**, 254-263, doi:<https://doi.org/10.1016/j.cej.2016.04.079> (2016).

5 Visual Minteq 2.32 v. 2.32. (KTH, Stockholm, Sweden, 2005).

6 The Geochemist`s Workbench - A User`s Guide to Rxn, Act2, React, and Gtplot v. Release 4.0 (University of Illinois, 2002).

7 Visual Minteq 3 v. 3 (KTH, Stockholm, Sweden, 2012).

8 Birch, W. D., Pring, A., McBriar, E. M., Gatehouse, B. M. & McCammon, C. A. Bamfordite, Fe3+Mo2O6(OH)(3)center dot H2O, a new hydrated iron molybdenum oxyhydroxide form Queensland, Australia: Description and crystal chemistry. *American Mineralogist* **83**, 172-177 (1998).

9 Wu, C. D. *et al.* Synthesis, structural characterization and properties of two new lamellar polymers: NH4H3Cu2Mo2O10 and KHFe2Mo2O10. *Journal of Alloys and Compounds* **368**, 342-348, doi:10.1016/j.jallcom.2003.08.075 (2004).

10 Schindler, M. & Hawthorne, F. C. Schubnelite, [Fe (super 3+) (V (super 5+) O 4 )(H 2 O)], a novel heteropolyhedral framework mineral. *American Mineralogist* **84**, 665-668, doi:10.2138/am-1999-0419 (1999).

11 Larsson, M. A., Persson, I., Sjöstedt, C. & Gustafsson, J. P. Vanadate complexation to ferrihydrite: X-ray absorption spectroscopy and CD-MUSIC modelling. *Environmental Chemistry* **14**, 141-150 (2017).

12 Peacock, C. L. & Sherman, D. M. Vanadium(V) adsorption onto goethite (α-FeOOH) at pH 1.5 to 12: a surface complexation model based on ab initio molecular geometries and EXAFS spectroscopy. *Geochimica et Cosmochimica Acta* **68**, 1723-1733, doi:<http://dx.doi.org/10.1016/j.gca.2003.10.018> (2004).

13 Brinza, L., Vu, H. P., Shaw, S., Mosselmans, J. F. W. & Benning, L. G. Effect of Mo and V on the Hydrothermal Crystallization of Hematite from Ferrihydrite: An in Situ Energy Dispersive X-ray Diffraction and X-ray Absorption Spectroscopy Study. *Crystal Growth & Design* **15**, 4768-4780, doi:10.1021/acs.cgd.5b00173 (2015).
